# Development of a Concussion Risk Function for a Youth Population Using Head Linear and Rotational Acceleration

**DOI:** 10.1007/s10439-019-02382-2

**Published:** 2019-10-28

**Authors:** Eamon T. Campolettano, Ryan A. Gellner, Eric P. Smith, Srinidhi Bellamkonda, Casey T. Tierney, Joseph J. Crisco, Derek A. Jones, Mireille E. Kelley, Jillian E. Urban, Joel D. Stitzel, Amaris Genemaras, Jonathan G. Beckwith, Richard M. Greenwald, Arthur C. Maerlender, Per Gunnar Brolinson, Stefan M. Duma, Steven Rowson

**Affiliations:** 1 Department of Biomedical Engineering and Mechanics, Virginia Tech, Blacksburg, VA USA; 2 Department of Statistics, Virginia Tech, Blacksburg, VA USA; 3 Department of Orthopaedics, The Warren Alpert Medical School of Brown University and Rhode Island Hospital, Providence, RI USA; 4 Virginia Tech-Wake Forest School of Biomedical Engineering and Sciences, Winston-Salem, NC USA; 5 Simbex, Lebanon, NH USA; 6 Center for Brain, Biology, and Behavior, University of Nebraska, Lincoln, NE USA; 7 Edward Via Virginia College of Osteopathic Medicine, Blacksburg, VA USA

**Keywords:** Biomechanics, Helmet, Risk curve, Mild traumatic brain injury, Football

## Abstract

Physical differences between youth and adults, which include incomplete myelination, limited neck muscle development, and a higher head-body ratio in the youth population, likely contribute towards the increased susceptibility of youth to concussion. Previous research efforts have considered the biomechanics of concussion for adult populations, but these known age-related differences highlight the necessity of quantifying the risk of concussion for a youth population. This study adapted the previously developed Generalized Acceleration Model for Brian Injury Threshold (GAMBIT) that combines linear and rotational head acceleration to model the risk of concussion for a youth population with the Generalized Acceleration Model for Concussion in Youth (GAM-CY). Survival analysis was used in conjunction with head impact data collected during participation in youth football to model risk between individuals who sustained medically-diagnosed concussions (*n* = 15). Receiver operator characteristic curves were generated for peak linear acceleration, peak rotational acceleration, and GAM-CY, all of which were observed to be better injury predictors than random guessing. GAM-CY was associated with an area under the curve of 0.89 (95% confidence interval: 0.82–0.95) when all head impacts experienced by the concussed players were considered. Concussion tolerance was observed to be lower for youth athletes, with average peak linear head acceleration of 62.4 ± 29.7 g compared to 102.5 ± 32.7 g for adults and average peak rotational head acceleration of 2609 ± 1591 rad/s^2^ compared to 4412 ± 2326 rad/s^2^. These data provide further evidence of age-related differences in concussion tolerance and may be used for the development of youth-specific protective designs.

## Introduction

As many as 1.9 million sports-related concussions occur annually in the United States for youth athletes below the age of 18.^4^ Recently, research has shown potential links between a history of concussions and long-term neurodegeneration.^34^,^35^,^51^ Ongoing development of the youth brain has been suggested as a factor in the heightened vulnerability of youth towards concussion.^22^ Concussive injuries in the youth population may also result in longer recovery times or even disrupt natural maturation of the brain, which makes the clinical diagnosis and management of particular concern.^22^,^26^

Numerous differences between adult and youth populations likely play a role in the increased susceptibility of youth towards concussion. Youth brains are still developing, with myelination not complete. Unmyelinated brain fibers have been shown to be more vulnerable to brain injury and recover more slowly than myelinated fibers, which lends credence to the differences in tolerance to concussion.^5^,^28^,^38^ Youth heads have grown to more than 90% of full-size by the age of five and reach full-size between the ages of 10 and 16.^11^,^33^ Body development lags behind the head, resulting in an increased head-body ratio for youths relative to adults. It is also known that children have reduced neck strength and musculature, with a limited capacity for mass recruitment to reduce resultant head acceleration.^7^,^14^,^20^,^22^,^28^ The unique aspects of the youth brain and its response to head impact and concussion necessitate the consideration of youth concussion as a distinct entity, and not just a scaled version of adult concussion.

Researchers have largely relied on head impact kinematic data collected from football players to model risk of concussion.^37^,^42^^–^44 This population is exposed to head impacts regularly and experiences concussions at a high rate among team contact and collision sports.^8^,^13^ These risk functions rely on linear and/or rotational head acceleration as predictors of concussion, as head kinematics are related to the brain’s inertial response. Reconstructions of concussive impacts in the National Football League led to the development of three concussion risk curves, though this dataset did not consider that most head impacts in football are subconcussive.^37^ As such, there is an overestimation of injury risk for acceleration inputs. The concussions in this dataset comprising professional football players were associated with peak head kinematics of 98 ± 28 g for linear acceleration and 6432 ± 1813 rad/s^2^ for rotational acceleration.^37^ Risk functions were also developed from on-field data generated using the Head Impact Telemetry System (HIT System, Simbex, Lebanon, NH) with high school and collegiate football players.^42^^–^44 These predictions considered estimates of concussion underreporting, as well as the relationship between concussive and subconcussive impacts. The concussions in the HIT System dataset comprising high school and collegiate football players were associated with peak head kinematics of 102 ± 33 g for linear acceleration and 4412 ± 2326 rad/s^2^ for rotational acceleration.^3^,^44^ The known differences between pediatric and adult populations preclude the use of these previously developed concussion risk functions for a youth population.

Injury data collected from head impact exposure studies provides in-depth biomechanical data on a subset of the youth football population. This study adapted an existing injury metric that combines linear and rotational head acceleration to model the risk of concussion for a youth population. For this study, the age range used to define youth was 9 to 14 years old, which represents the ages associated with athletes participating in tackle football below the high school level. The predictive capacity of this injury metric was compared to previously used biomechanical parameters. We hypothesized that youth athletes would have a lower tolerance for concussion than adult athletes.

## Methods

A large cohort (*n* = 124) of youth football players at three sites (Brown University, Virginia Tech, and Wake Forest University) between the ages of 9 and 14 received helmets instrumented with accelerometer arrays (HIT System). This study was approved locally by each university’s institutional review board and parental consent was obtained for each athlete, with athletes providing verbal assent independently. More than 400 total player-seasons of head impact data were collected from the 2015 season through the 2018 season, and the cohort of 124 players was a representative sample of the overall study population.

The HIT System accelerometers are mounted on an elastic base in order to maintain contact with the head throughout impact, which allows the measurement of head acceleration rather than helmet acceleration.^25^ Head impact data consisted of peak linear and rotational head acceleration values. Only data from head impacts with a resultant linear acceleration exceeding 10 g were included. This 10 g threshold differentiates between acceleration levels associated with impact and non-impact events.^31^

Concussion diagnoses were made by clinicians at each site through clinical examination in addition to objective assessment measures. All diagnoses followed the guidelines set forth by the Fifth International Conference on Concussion in Sport.^27^ Some athletes were immediately removed from competition due to experiencing a head impact that was associated with their concussion, whereas other athletes experienced a delayed onset of symptoms that was disclosed later in the competition or at its conclusion. Through interviews with the injured athletes, as well as video review of the playing session in which the injury occurred, we were able to associate each athlete’s injury with a specific head impact.

Underreporting of concussions is a known issue, though the youngest age group for which these data exist is the high school level. Athletic trainers reported 5% of athletes sustain a concussion, while 47% of high school players report sustaining a concussion on surveys that do not include the word “concussion”.^16^,^24^ While the underreporting rates for youth athletes are unknown, it is likely that some subset of players who were instrumented in our study sustained a concussion but failed to report it or seek medical attention. For this reason, only head impact data from athletes who sustained a clinically-diagnosed concussion were included in this analysis. These athletes sought medical attention, so we have no reason to believe that these subjects would not report other injuries. By not including head impact data from all instrumented athletes, our analysis represents a more conservative assessment of concussion risk for a vulnerable subset of players. A total of 11 youth athletes in our cohort sustained medically-diagnosed concussions for which corresponding head impact data were also available. To increase our sample size, concussive head impact data (*n* = 4) were used from previously published work in which youth football athletes within the study age range received helmets instrumented with HIT System accelerometer arrays.^6^,^9^ In total, head impact data from 15 players who sustained concussions as a result of participation in youth football were included in this analysis. It should be noted that determination of injury prevalence would have to consider the total player sample, and not just the specific study cohorts. Each athlete’s head impact exposure history for the season in which the injury occurred was included, with head impacts being coded as concussive or non-concussive.

### Youth Concussion Risk Function

Rather than attempting to fit a cumulative distribution function to the bivariate head impact data, the peak linear and rotational head acceleration values for each concussive head impact were combined into an overall measure of magnitude. This aggregate measure was modeled after Generalized Acceleration Model for Brain Injury Threshold (GAMBIT),^29^,^30^ which was developed to consider the combined effect of linear and rotational kinematics in the presentation of brain injury. GAMBIT considered more serious brain injuries than concussion, so the critical values in the original equation were modified here to be relevant to our injury severity and youth population. The Generalized Acceleration Model for Concussion in Youth (GAM-CY) is given by

1 $${\text{GAM - CY}} = \sqrt {\left( {\frac{\text{PLA}}{{{\text{PLA}}_{{{\text{conc avg}} .}} }}} \right)^{2} + \left( {\frac{\text{PRA}}{{{\text{PRA}}_{{{\text{conc avg}} .}} }}} \right)^{2} }$$

where ${\text{PLA}}$ and ${\text{PRA}}$ are peak linear acceleration and peak rotational acceleration respectively, and ${\text{PLA}}_{{{\text{conc avg}} .}}$ and ${\text{PRA}}_{{{\text{conc avg}} .}}$ are critical values corresponding to the average peak head kinematics associated with the 15 concussive impacts in this study.

A modified form of survival analysis was used to develop an injury risk curve that considered GAM-CY as a predictor of concussion. Recently, it has been suggested that concussion tolerance varies between individuals, and that aggregate analysis may not be the most effective way to model this injury.^46^ Rather than modeling individual head impacts as inputs to determine risk, Kaplan–Meier curves were developed for each individual athlete’s head impact history. Kaplan–Meier estimators retain a 0 value for non-injurious measurement levels. For injurious levels, the estimator is calculated as having a value equal to the probability of sustaining a concussion for all head impacts sustained at the injurious level or greater.^21^ For example, if an athlete sustained 9 head impacts with magnitudes exceeding that of his concussive head impact, the Kaplan–Meier estimator would have a value of 0.1, or 10%, as 1 in 10 head impacts resulted in injury at that level. Individual risk values at kinematic levels associated with concussion were then averaged across players and fit to a cumulative distribution function. This approach towards calculating risk resulted in players with different head impact histories contributing equal weighting towards the resulting risk function.

Each of the 15 Kaplan–Meier curves was defined only for the range of GAM-CY values over which the player experienced a head impact, and the point at which individual risk became non-zero was at the concussive GAM-CY value for that specific player. To generate a single, composite risk curve, the risk values at each of the concussive GAM-CY values were averaged across players. Only players with impacts as severe as the concussive impact were considered in the average. For example, only two players in this dataset experienced an impact with a GAM-CY value as severe as the hardest concussion. Average risk at that severity was only computed considering those two players.

A log-normal distribution was then fit to the average risk values computed for magnitudes of GAM-CY associated with concussion. The log-normal cumulative distribution function takes the form of Eq. (2), with x representing GAM-CY, $\mu$ as the distribution mean, and $\sigma$ as the distribution standard deviation. Though direct calculation of the probability is complicated by the presence of the error function, most software packages have built-in functionality to complete this calculation (MATLAB: *logncdf*; Microsoft Excel: *lognorm.dist*; R: *plnorm*). Log-normal parameters were estimated using a least-squares technique.

2 $$\ln_{\text{cdf}} = \frac{1}{2} + \frac{1}{2}erf\left[ {\frac{\ln \left( x \right) - \mu }{\sqrt 2 \sigma }} \right]$$

### Youth Concussion Risk Function Confidence Interval

Uncertainty associated with player sampling was modeled by resampling individual Kaplan–Meier curves 10,000 times. These bootstrapped samples were then used to generate 10,000 log-normal curve fits with parameters estimated in a manner identical to the risk curve developed using the measured injury data. At each value of GAM-CY, the 95% confidence bounds were determined by taking the 250th and 9750th ranked values.

### Receiver Operator Characteristic Curve Analysis

The combined biomechanical parameter, GAM-CY, was compared against linear and rotational head acceleration for its predictive capability.^43^ The predictive capability was assessed by computing the area under the receiver operator characteristic (ROC) curve (AUC). For comparison, random guessing would be associated with an AUC equal to 0.5. Direct comparison of AUC for each parameter was conducted using Hanley’s method.^17^,^18^ A significance level of 0.05 was used for all statistical tests.

### Comparison to Other Populations

The biomechanics of concussive head impacts for the youth athletes in this study were compared to what has been previously published for high school and collegiate athletes.^44^ This previous work made use of the HIT System and collected head impact data for 57 instances of medially-diagnosed concussions. The mean values associated with concussion for the two athlete groups were compared for linear and rotational head acceleration using Welch’s *t* test.

By only including the subset of youth athletes who sustained a clinically-diagnosed concussion in the dataset, the potential existed that this group of players would not be representative of all youth athletes instrumented in terms of head impact exposure. To relate the head impact exposure profiles of the concussed and non-concussed youth athletes, we computed the 95th percentile GAM-CY value and risk-weighted exposure for each athlete (*n* = 124). The 95th percentile GAM-CY value is a measure of the severity of an athlete’s head impact profile, with a higher 95th percentile value being associated with more severe, or higher risk, head impacts. As most football head impacts are associated with lower magnitude accelerations, there is greater variance between athletes for 95th percentile values than for median values. Further, 95th percentile values are more representative of the magnitudes for head impacts typically associated with injury.^32^,^42^ Risk-weighted exposure is an aggregate measure that combines impact frequency and magnitude as a means of considering overall head impact exposure.^12^,^40^,^42^,^53^ The risk of concussion was computed for each head impact sustained by an athlete, and then these individual risk values were summed together into one measure. The cohort distributions (concussed vs. non-concussed) were compared using a Wilcoxon Ranked Sum test, and effect size was determined using Cohen’s *d.*

## Results

**Figure 1:**
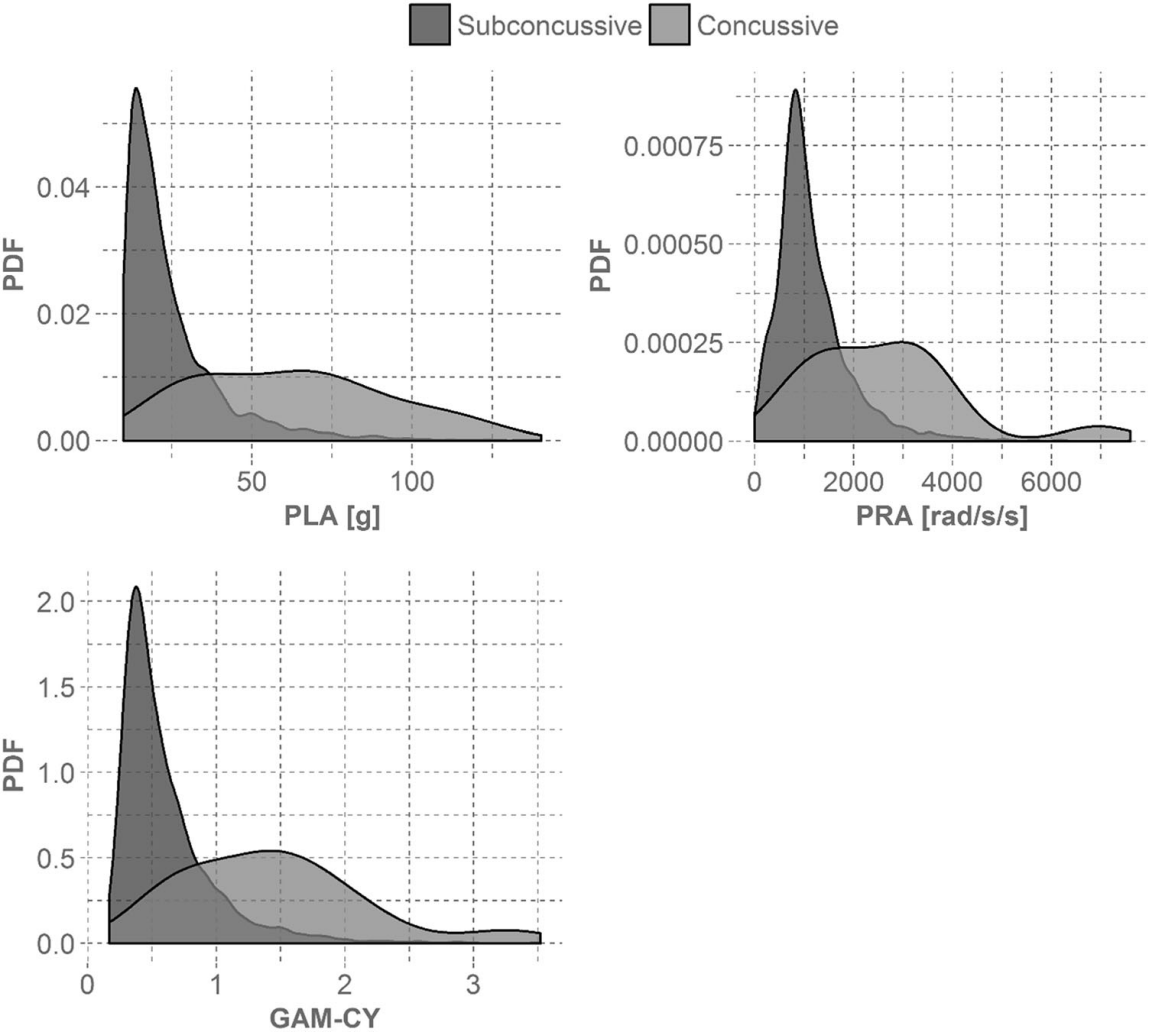
Empirical probability density function (PDF) of subconcussive and concussive head impacts. The distribution of subconcussive head impacts was heavily right-skewed, while the distribution of concussive head impacts was less well-defined. The median peak acceleration values were 19.5 g and 970 rad/s^2^ for the subconcussive head impacts and 63.8 g and 2599 rad/s/s for the concussive head impacts. The median GAM-CY value for subconcussive head impacts was 0.49 and 1.36 for concussive head impacts.

**Figure 2:**
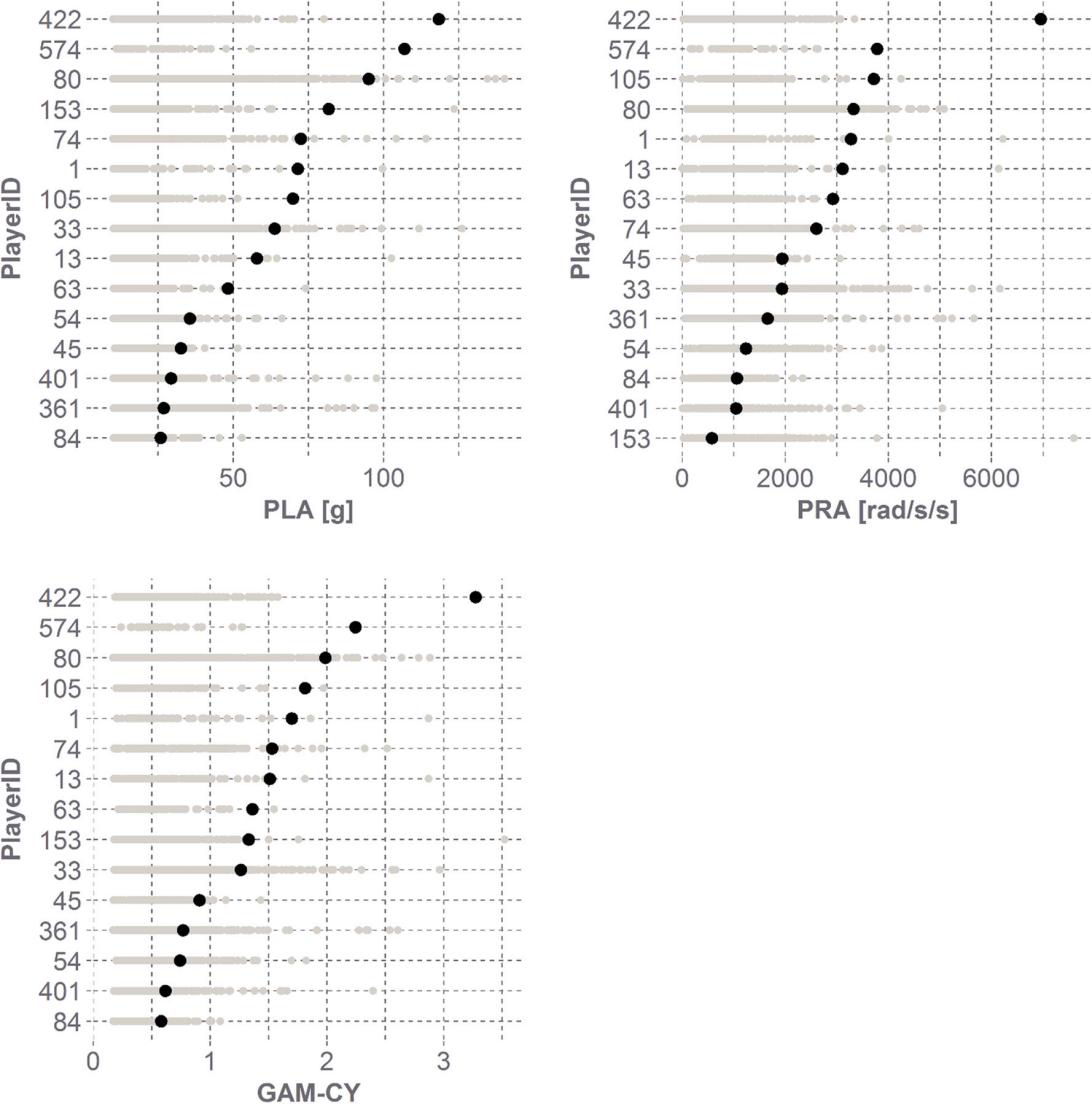
60% of concussive head impacts were among a player’s top 10 hardest head impacts. All concussive head impacts were within the top quartile of a player’s head impacts when considering the combination of linear and rotational kinematics.

The 15 players in this study experienced a total of 3757 head impacts in the season in which they sustained their concussion, with a median and 95th percentile linear head acceleration of 19.5 and 57.1 g and a median and 95th percentile rotational head acceleration of 972 and 2593 rad/s^2^ (Fig. 1). Peak linear and rotational head acceleration values associated with concussion varied among the athletes in this study (Table 1). The average concussive head impact was associated with a peak linear head acceleration of 62.4 ± 29.7 g and a peak rotational head acceleration of 2609 ± 1591 rad/s^2^. For most athletes, the concussive head impact was among the top 10% of all head impacts experienced by that athlete for that season (Fig. 2). Based on the impact locations designated by the HIT System, it was observed that six of the 15 concussions were due to impacts to the front of the helmet, five to the back of the helmet, and two each for the top and sides.^15^

**Table 1:** Biomechanical summary of player concussions.

PlayerID	Impacts	PLA [g]	PRA [rad/s^2^]	GAM-CY	Rank in PLA	Rank in PRA	PLA percentile	PRA percentile
1	61	71.5	3272	1.70	2	3	98.3	96.7
13	159	57.9	3112	1.51	5	3	97.5	98.7
33	579	63.8	1936	1.26	26.5	113	95.6	80.7
45	163	32.6	1938	0.91	8.5	9	95.4	95.1
54	322	35.6	1238	0.74	20	86	94.1	73.6
63	113	48.2	2922	1.36	2	1	99.1	100.0
74	223	72.5	2599	1.53	7	10	97.3	96.0
80	393	95.1	3324	1.99	10	31	97.7	92.4
84	107	25.9	1061	0.58	22	30	80.4	72.9
105	93	69.9	3716	1.81	1	2	100.0	98.9
153	208	81.8	578	1.33	2	172	99.5	17.8
361	616	26.9	1658	0.77	168	92	72.9	85.2
401	228	29.3	1047	0.62	44.5	69	80.9	70.2
422	452	118.4	6955	3.27	1	1	100.0	100.0
574	40	106.9	3781	2.24	1	1	100.0	100.0

While peak linear acceleration (PLA) and peak rotational acceleration (PRA) values varied for each concussion, most concussions were associated with some of the athletes’ hardest head impacts. Ranks were determined in descending order, with the highest PLA or PRA value being associated with a rank of 1.

### Youth Concussion Risk Function

**Figure 3:**
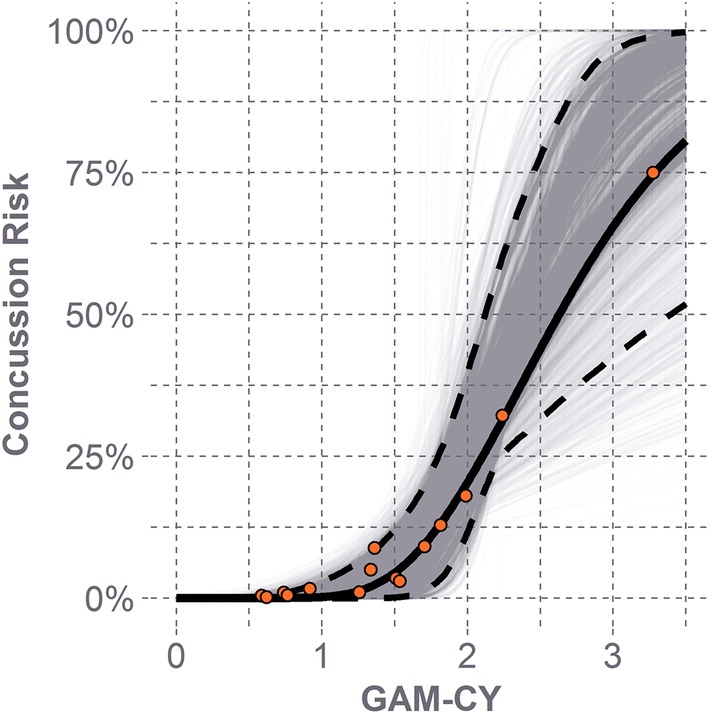
Log-normal distribution fit to concussion data (solid line) with 95% confidence bounds (dashed lines). Most concussive head impacts were associated with lower values of GAM-CY. Fewer concussive head impacts were observed for higher values of GAM-CY (> 2), so the 95% confidence bounds are much wider at these values. Gray lines represent log-normal distributions fit from the bootstrap samples for the concussion data.

**Figure 4:**
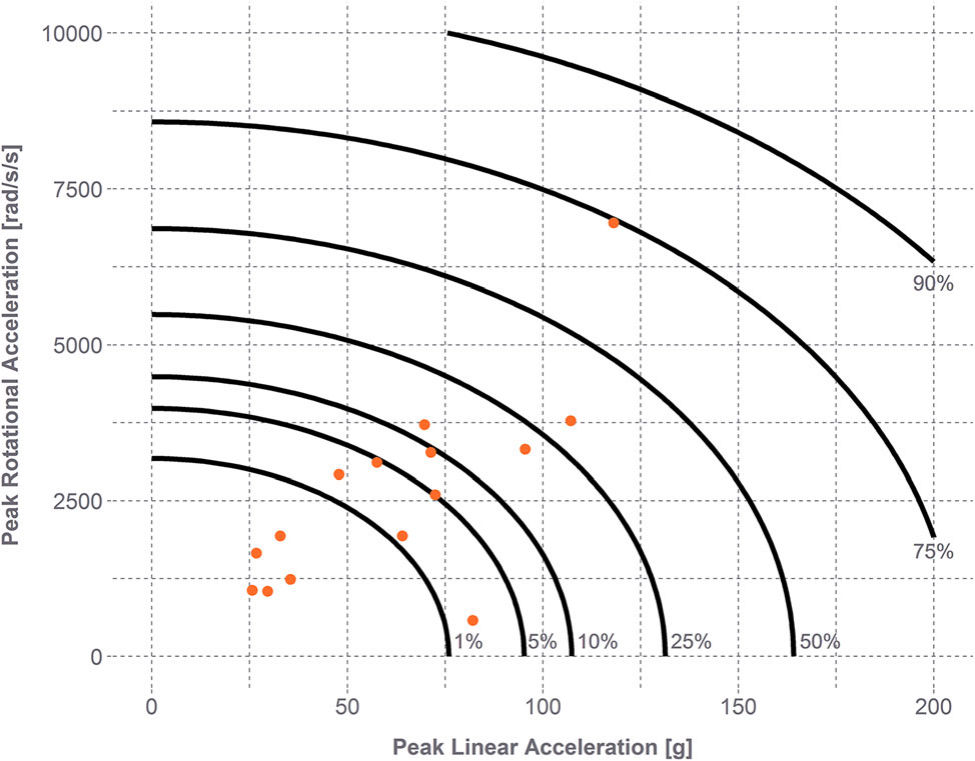
Risk of concussion as a function of linear and rotational head acceleration. Most concussive head impacts were associated with average risk of concussion below 20%.

The peak linear and rotational head acceleration values for the concussive impacts were used to compute GAM-CY. The average CDF relating GAM-CY to risk of concussion was fit to a log-normal distribution with the following parameters: $\mu$ = 0.967 and $\sigma$ = 0.331 (Fig. 3). These risk values were then related back to peak linear and rotational head acceleration (Fig. 4).

3 $${\text{GAM - CY}} = \sqrt {\left( {\frac{\text{PLA}}{62.4}} \right)^{2} + \left( {\frac{\text{PRA}}{2609}} \right)^{2} }$$

4 $${\text{Concussion risk}} = \frac{1}{2} + \frac{1}{2}{\text{erf}}\left[ {\frac{{\ln \left( {\text{GAM - CY}} \right) - 0.967 }}{\sqrt 2 \times 0.331}} \right]$$

### Youth Concussion Risk Function Confidence Interval

Given the paucity of concussive data points at the higher end of linear and rotational head acceleration values, there is greater uncertainty in the confidence interval for the risk function at higher biomechanical values (Fig. 3). Nearly all (13 of 15) of the concussive head impacts occurred at GAM-CY values below 2. Below this value, the confidence bounds were observed to be much narrower.

### Receiver Operator Characteristic Curve Analysis

**Figure 5:**
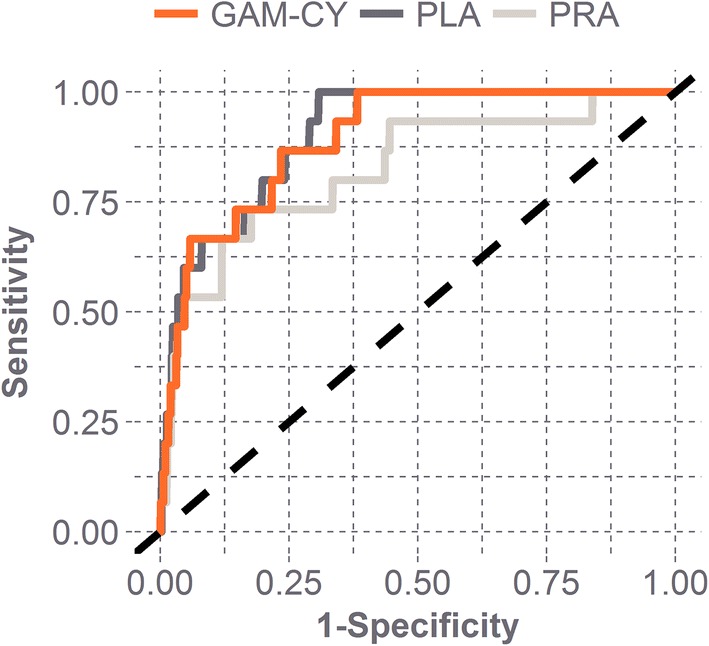
ROC curves for GAM-CY, PLA, and PRA. All parameters were significantly better than random guessing (dashed line), with peak rotational acceleration (PRA) offering the least predictive capability among all metrics.

ROC curves were generated to assess the predictive capacity of GAM-CY, peak linear acceleration, and peak rotational acceleration (Fig. 5). All of these metrics were found to be better predictors of concussion than random guessing for this dataset (*p *<0.05). No significant difference was observed between GAM-CY and any of the other metrics assessed in this study (Table 2).

**Table 2:** AUC for ROC curves.

Metric	AUC	95% CI	*p*-value RG	p-value GAM-CY
PLA	0.904	0.842–0.951	< 0.0001	0.462
PRA	0.824	0.662–0.918	< 0.0001	0.267
GAM-CY	0.894	0.818–0.947	< 0.0001	–

*p*-value compared to random guessing is denoted as *p*-value RG. *p*-value compared to GAM-CY is denoted as *p*-value GAM-CY. All measures offer better predictive capacity than random guessing

### Comparison to Other Populations

It was observed that the youth athletes in this study experienced concussions at biomechanical levels that are lower than what has been reported for high school and collegiate football players.^44^ The youth athletes in this study experienced concussive head impacts associated with average peak linear head acceleration values of 62.4 ± 29.7 g, which is lower than the 102.5 ± 32.7 g recorded for high school and collegiate players (*p *= 0.0001 and *d* = 1.247). This difference between populations was consistent for rotational acceleration, with average peak rotational head acceleration values of 2609 ± 1591 rad/s^2^ for youth athletes compared to 4412 ± 2326 rad/s^2^ for the high school and collegiate athletes (*p *= 0.001 and *d* = 0.82).

**Figure 6:**
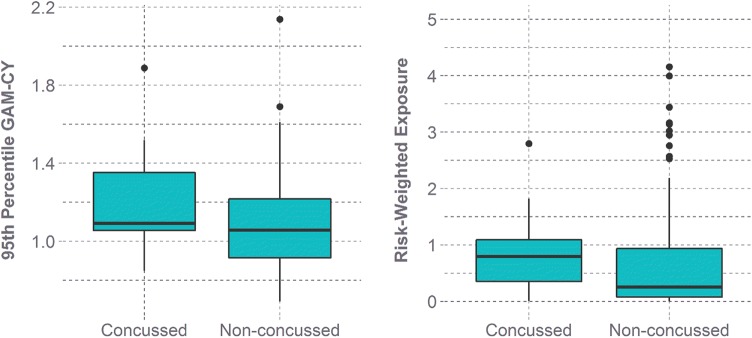
Comparing concussed athletes to non-concussed athletes. The median values for the 95^th^ percentile GAM-CY and risk-weighted exposure were higher for the concussed cohort than for the non-concussed cohort.

The non-concussed sample used to compare to the head impact exposure profiles of the 15 athletes who sustained a clinically-diagnosed concussion in this study consisted of 113 unique youth athletes. There was evidence of athletes who sustained a concussion having higher 95th percentile GAM-CY values (*p* = 0.106 and *d* = 0.516) and risk-weighted exposure (*p *= 0.052 and *d *= 0.785) than their non-concussed counterparts. The head impact exposure distribution for athletes who did not sustain a concussion was associated with a larger range of risk-weighted exposure values (Fig. 6).

## Discussion

This study adapted a previously-developed brain injury metric that relates peak linear and rotational head acceleration for use with a youth population to model concussion risk. The consideration of both linear and rotational kinematics stems from the fact that both likely contribute in the development of concussion and are associated with different injury mechanisms.^19^,^23^,^36^,^52^ Linear kinematics are best correlated with an induced intracranial pressure gradient while rotational kinematics are best correlated with the brain’s strain response. This injury metric was developed from youth head impact data collected using the HIT System. This injury metric builds on previous injury assessment efforts but was uniquely developed towards a youth population.^29^,^30^,^43^ Only head impact data from youth athletes who sustained a concussion were included in the development of the present injury metric. This represented a more conservative approach to injury risk while also providing for higher confidence in classifying concussive and subconcussive head impacts. A subset of our instrumented athletes who did not report concussion symptoms or seek medical attention likely sustained concussions due to participation in football. Despite a recent focus on concussion education, underreporting still remains a factor even in youth football.^39^ The injury metric presented here was modeled after GAMBIT, though the critical values for linear and rotational head acceleration were based on the average values associated with concussion and not on more serious brain injuries which have been observed in cadaver testing.^30^ The average values for linear and rotational head acceleration associated with concussion represented the 96th percentile of head impacts experienced by youth athletes in this dataset.

### Individualized Nature of Concussion

For most athletes, their concussive impact was among the top 10% most severe head impacts they experienced for the season in which they sustained their concussion. When considering all the head impacts in the dataset independent of which player sustained them, some of these concussive head impacts would appear to be less severe. This way of assessing risk would be consistent with how previous concussion risk curves have been developed, in that head impacts are considered in aggregate.^37^,^43^,^44^ Recently, concussion tolerance has been presented as being specific to an individual. It was observed that 90% of concussive impacts for high school and collegiate football players occurred at levels within an individual athlete’s top 5 highest magnitude impacts.^46^ By developing Kaplan–Meier curves for each athlete in this study who sustained a concussion, we retained the severity of the concussive head impact for the individual while also considering the variance between our subjects. These individual risk curves normalized each player’s head impact history so that a composite risk curve could be generated using all of the injured players. This normalization process ensured that each player contributed equally towards the development of the overall risk function regardless of the number of head impacts each player experienced.

#### Youth Concussion Risk Function Confidence Interval

For higher values of GAM-CY, where there are fewer concussive head impacts and fewer overall head impacts, there is much greater uncertainty in concussion risk estimated by the risk function developed here (Fig. 3). A much narrower confidence interval is observed at lower values of GAM-CY, where more head impacts, both concussive and subconcussive, occurred. With a greater number of concussions, particularly at higher levels of GAM-CY, one would expect that the confidence in the fit would increase. Nearly all of the head impacts (97%) recorded on the field were associated with GAM-CY values below 1.5, where there is greater confidence in the estimated risk. This risk function was developed with the target application of evaluating the relative effectiveness of youth football helmets at reducing energy transfer to the head. As helmet testing protocols should ideally be representative of the actual impact scenarios that players would experience on the field, it can be expected that the test conditions would be within this higher confidence area of the risk curve.

#### Receiver Operator Characteristic Curve Analysis

Peak head impact kinematics have shown to be good predictors of concussion, with increases in head acceleration leading to increased injury risk.^3^,^10^,^42^ This was also observed for each of the predictors investigated in this study. Rotational acceleration was observed to have the lowest predictive capacity (AUC = 0.824 [95% CI 0.662–0.918]) of all predictors. Peak linear acceleration (AUC = 0.904 [95% CI 0.842–0.951]) and GAM-CY (AUC = 0.894 [95% CI 0.818–0.947]) were associated with similar AUCs and can predict the concussions in this dataset equally well. Most head impacts in football are similar, with impacts to the front, side, and back of the helmet having similar relationships between linear and rotational head acceleration. Impacts to the top of the head, though, often result in very low values of rotational head acceleration despite a wide range of linear acceleration values. The lower predictive capacity for rotational acceleration alone is thus expected. Peak linear acceleration, though a strong predictor of concussion, does not consider rotational kinematics. With concussion being an injury likely related to both linear and rotational kinematics, an approach with combined kinematics seems the most viable.

#### Sensitivity Analysis

To investigate the potential effect of additional concussive data points changing the critical values used to compute GAM-CY, a sensitivity analysis was conducted. The critical values for peak linear acceleration and peak rotational acceleration were lowered or raised by 25% and the ability of these modified forms of GAM-CY to differentiate concussive and subconcussive impacts was assessed using the area under the ROC curves. All combinations of decreased and increased linear and rotational critical values were considered, and it was observed that GAM-CY was insensitive to changes in critical values. AUC values varied from 0.882 to 0.901, compared to the presented value of 0.894.

#### Comparison to Other Populations

The average kinematic values associated with youth concussion (PLA: 62.4 ± 29.7 g and PRA: 2609 ± 1591 rad/s^2^) are much lower (*p *< 0.001 and *d* > 0.82) than what has been reported for high school and college athletes (PLA: 102 ± 33 g and PRA: 4412 ± 2326 rad/s^2^) and professional football players (PLA: 98 ± 28 g and PRA: 6432 ± 1813 rad/s^2^).^37^,^44^ Some of the concussive head impacts for youth athletes in this study exceeded the severity of the average concussion for the older populations considered. Based on the measured differences in concussive kinematics between the two populations and the known physical differences, data from this study support the hypothesis that the youth population has a lower biomechanical tolerance for concussion.

The distributions between the two youth cohorts (concussed vs. non-concussed) had considerable overlap for both 95th percentile GAM-CY and risk-weighted exposure, though median values were greater for the concussed cohort. On average, the athletes who experienced clinically-diagnosed concussions experienced more severe head impact exposure profiles than their non-concussed counterparts, though variability between subjects cannot be understated. Athletes who sustained clinically-diagnosed concussions did not participate in practices and games during their recovery process. This may partially explain why there is a higher range of risk-weighted exposure values for the athletes who did not sustain a concussion (Fig. 6).

### Implications for Safety Equipment

As helmet manufacturers continue to refine and design technologies to mitigate linear and rotational head acceleration during head impacts, the risk function developed here will be a useful tool in evaluating the effectiveness of these changes. While the evaluation of football helmets will likely be the primary application of this risk function, the potential exists to expand into other industries, such as automotive and other forms of head protection. Provided the impact profiles are similar to football head impacts, the development of this concussion risk function can be used to help the development of safer products aimed at limiting the potential for concussion.^45^,^47^,^48^

#### Limitations

This analysis was limited by several factors. The developed injury metric was based on only data from 15 youth football players who sustained concussions. While a limited sample size, this represents the largest repository of youth injury data. Continued data collection may lead to further refinement of this injury metric. By only using data from concussed athletes and not considering underreporting, a more conservative approach of injury risk is achieved. In the interest of evaluating the safety of football helmets and potentially other protective equipment, overestimating injury risk is more favorable than underestimating injury risk. This function considered only the effects of single impacts, as its designed application is towards the evaluation of the effectiveness of football helmets in mitigating energy transfer to the head. It is also likely that other factors, such as impact location, impact duration, exposure to repetitive head impacts, and biological factors contribute towards injury risk.^2^,^3^,^41^,^50^ The brain’s susceptibility to injury varies by the direction of force of the impact, and continued data collection may provide greater variety in concussive head impact locations which would allow for the consideration of impact location in the risk of concussion. The HIT System has known measurement error uncertainties associated with single impact measurements, though these uncertainties can vary by impact location and coupling between the head and the accelerometer array.^1^,^49^ Rotational velocity correlates best with the brain strain response associated with concussion but is not measured by the HIT System.^19^ Only peak linear and rotational head acceleration were used to estimate risk of concussion, though these parameters were found to have good injury prediction capabilities. These limitations must be considered when attempting to use our injury metric to evaluate the risk of concussion for a youth population.

## Conclusion

This study presents a concussion risk function for a youth population based on peak linear and rotational head acceleration from a single head impact. Concussive and subconcussive head impact data from youth football players who sustained a concussion were used to determine the injury metric with the highest predictive capability. While ROC analysis revealed that all parameters were good predictors of concussion, rotational acceleration was shown to be the least predictive. GAM-CY is highly predictive of concussion (AUC = 0.894) and considers both linear and rotational head kinematics, in addition to being specific to a youth population. Concussions within the youth population were associated with lower biomechanical values than what has previously been observed for adults. Helmet manufacturers and automotive companies may develop safer products by utilizing this risk function.
